# Statistical methods used in the public health literature and implications for training of public health professionals

**DOI:** 10.1371/journal.pone.0179032

**Published:** 2017-06-07

**Authors:** Matthew J. Hayat, Amanda Powell, Tessa Johnson, Betsy L. Cadwell

**Affiliations:** 1 School of Public Health, Georgia State University, Atlanta, GA, United States of America; 2 Department of Sociology, Georgia State University, Atlanta, GA, United States of America; 3 Center for Surveillance, Epidemiology and Laboratory Services, Centers for Disease Control and Prevention, Atlanta, GA, United States of America; Hunter College, UNITED STATES

## Abstract

Statistical literacy and knowledge is needed to read and understand the public health literature. The purpose of this study was to quantify basic and advanced statistical methods used in public health research. We randomly sampled 216 published articles from seven top tier general public health journals. Studies were reviewed by two readers and a standardized data collection form completed for each article. Data were analyzed with descriptive statistics and frequency distributions. Results were summarized for statistical methods used in the literature, including descriptive and inferential statistics, modeling, advanced statistical techniques, and statistical software used. Approximately 81.9% of articles reported an observational study design and 93.1% of articles were substantively focused. Descriptive statistics in table or graphical form were reported in more than 95% of the articles, and statistical inference reported in more than 76% of the studies reviewed. These results reveal the types of statistical methods currently used in the public health literature. Although this study did not obtain information on what should be taught, information on statistical methods being used is useful for curriculum development in graduate health sciences education, as well as making informed decisions about continuing education for public health professionals.

## Introduction

Public health practice relies on the peer reviewed public health literature for current research and findings that support an evidence basis for effective practice. Studies have shown that statistical literacy and knowledge are needed for understanding published research [1]. The rapid growth and widespread availability in computing power and user-friendly statistical software packages in recent decades has led to the use of more advanced statistical methods and analyses being used and reported in the health literature [2]. However, statistical training in public health may not have kept up with the modern data explosion and statistical complexities increasingly being applied in health studies and reported in scientific publications. A comprehensive understanding of statistical concepts and methods is essential for understanding current research and developing effective public health practice.

Biostatistics education is a core requirement in all graduate degree public health programs accredited by the Association of Schools and Programs of Public Health (ASPPH) in the United States [3]. One of the core curriculum competencies in biostatistics education for the master of public health (MPH) degree is to develop skill and knowledge to critically evaluate the application, presentation, and interpretation of statistical analyses in public health studies [3,4,5]. Although this is a desired outcome of training, there are no known recent studies that quantify the types of statistical methods used in the public health literature. Information on methods used is needed to make informed decisions about curriculum development, continuing education, and training of public health professionals.

The purpose of this work is to quantify the use of basic and advanced statistical methods in the general public health literature. A critical question of interest is “What statistical concepts and methods do public health professionals need to know to read and understand the literature?” Our study provides the needed evidence basis for beginning to answer this question.

## Methods

The data collection form used in this study was created by the study authors and designed to gather information on statistical methods described in each randomly selected article. The form was rigorously developed and tested prior to use on the study sample. Our data collection form consisted of a closed-coding system for quantifying statistical methods reported by the authors of each published paper. We developed the form through a process of four pilot studies in which three reviewers read and cataloged the statistical methods reported in random samples of articles from our selected journals for the 2014 publication year. The results presented in the following tables in this article are framed to correspond to the data collection form. In reviewing each article, the selection of any variable on the review form indicated that variable had been explicitly or implicitly reported within the text of the paper. The final form included domains with specific items in each for article type, study design, sampling technique, summary statistics, reporting of statistical inference, statistical tests, statistical models, reporting of missing data, causal inference, and statistical software.

We aimed to obtain a list of influential general public health journals from which to sample articles. We sampled articles from seven top tier public health journals using the following method. Journals were selected based on a multi-faceted process. First, to gauge a general familiarity with general public health journals, we conducted an online internet search using the term “most influential public health journals.” From this, we compiled a master list of fourteen journals appearing on three or more lists identified from our online search. Next, one of the authors informally surveyed three experienced public health faculty members for suggestions of reputable public health journals. None were added, as all journals suggested were on our list. We next checked that all journals were recognized and included in PubMed. “PubMed comprises more than 26 million citations for biomedical literature from MEDLINE, life science journals, and online books.” (pubmed.com)” We next examined impact factors, deciding in advance to only include journals with exceptional impact factors. The cutoff was set at 3.0. After eliminating journals that were deemed more medically-focused and those that were specific to public health topics (e.g., policy, environmental health), we had seven remaining journals. Table 1 displays the 5-year impact factors for the 7 selected journals. The lowest impact factor was 4.245 for the *European Journal of Epidemiology* which we agreed was acceptable. We considered this set of 7 journals to comprise a representative sample of the top-tier general public health literature.

**Table 1:** Public health journals reviewed and number of articles from 2013 included in the study sample.

Journal	Sections included	5-year Impact Factor (as cited in 2013)	Eligible Articles	Study Sample	
				Proportion by Journal	Sampled Articles
			*#*	*%*	*#*
*Epidemiology (EPI)*	All substantive research articles	6.894	308	30.1	64
*American Journal of Epidemiology (AJE)*	*Original Contributions*, *Practice of Epi*, *Systematic Reviews & Pooled Analyses*	6.067	296	29.0	62
*American Journal of Public Health (AJPH)*	*Research and Practice*, *Online Research and Practice*	4.997	141	13.8	30
*Bulletin of World Health Organization (BWHO)*	*Research*	5.086	79	7.7	16
*European Journal of Epidemiology (EJE)*	All substantive research articles	4.245	75	7.3	16
*American Journal of Preventive Medicine (AJPM)*	*Research Articles*, *Review and Special Articles*	5.092	62	6.1	14
*International Journal of Epidemiology (IJE)*	All substantive research articles	8.000	62	6.1	14
**Total**			**1023**	**100**	**216**

### Sample size determination

The goal of this study was to quantify the types and frequencies of use of statistical methods in the public health literature. For purposes of determining the number of articles to be sampled to adequately accomplish this, we considered statistical methods that were rarely used. Thus, we concluded that if we calculated the sample size needed to detect rarely used methods, we would have a sufficient sample size to also cover the other more frequently occurring methods. Some advanced statistical techniques (such as nonlinear regression) were reported in only 1 of 42 articles in our pilot work. We therefore used ≈2.4% (= 1/42) as our estimate for a rarely used method to determine sample size with a precision estimation approach. Detection of a proportion of occurrence of 0.024 for an infrequently occurring statistical method and a given precision (interval width) of 0.05 resulted in a needed sample size of 188 articles. This is a reasonable precision within which we can be confident in our detection of rarely used statistical methods in the public health literature. We equated the notion of ‘attrition’ in our study to inappropriate articles that we agreed should be excluded from review (e.g., qualitative studies, editorials, etc.). In our pilot work, we had 5 articles appearing in the Research section of the journal that we deemed unsuitable for review. This equated to an ‘attrition’ rate of ≈12% (5/42). Assuming an attrition rate of 12%, we estimated a target sample size of 211 articles. Since we had 4 reviewers, for purposes of rounding, we decided to sample a total of 216 articles

### Sample

The journals selected were *American Journal of Public Health*, *American Journal of Preventive Medicine*, *International Journal of Epidemiology*, *European Journal of Epidemiology*, *Epidemiology*, *American Journal of Epidemiology*, *and Bulletin of the World Health Organization*. All research based articles published in 2013 in these journals were eligible for review. Table 1 displays the names of our selected study journals, descriptions of the sections from which articles were sampled, and the number of eligible and sampled articles. There were a total of 1,023 research articles published in total across all seven study journals.

### Data collection and analysis

We randomly sampled with probability proportional to the number of articles contributed by each journal [S1 File]. The 216 articles that comprised the study sample were then randomly allocated to four article groupings of 54 articles each. Each of the four reviewers was randomly assigned to review two of these 54-article groups (for a total of 108 articles per reviewer) and paired with one other reviewer for each article group. Review pairs consisted of one senior author (BC, MH) and one junior author (AP, TJ), such that both senior authors worked with both junior authors but not with one another and vice versa. Reviewers read and cataloged each article individually and a final consensus was reached in review pairs. Each pair met to review their assigned articles in three waves (wave 1 = 15 articles, wave 2 = 19 articles, and wave 3 = 20 articles), and the ordering of review and discussion between pairs was randomly ordered to mitigate learning and other group interaction effects on data collection. All four reviewers met as a large group periodically throughout the review process to discuss flagged articles and to ensure procedural consistency. Criteria for flagging articles included articles questionable for inclusion in our study (e.g., qualitative studies, program evaluation, study design overview reports).

Data entry was conducted using EpiInfo7 [6] and data analysis performed with the SAS Software System (SAS Institute, Cary NC). The online database created in EpiInfo7 was designed to match the paper form used throughout the review process for ease of data entry and efficiency. Master copies of the paper forms drafted during each pair reviewer meeting were collected and hand-entered by one of the reviewers. Upon completion of data entry, 10% of the records were randomly sampled, and a second reviewer cross-checked the entered records with the master copies. Percent agreement was near 100%, indicating a high confidence with the accuracy of the data entry process.

Data analysis consisted of frequency distributions for all study variables.

## Results

A total of 216 articles were reviewed. Table 2 displays the frequency of reported study types, as well as occurrence of descriptive and inferential statistics. The majority of articles were substantively focused (93.1%, n = 201) and reported an observational study design (81.9%, n = 177). Descriptive statistics (91.7%, n = 198) and tables (95.4%, n = 206) were reported in the vast majority of articles. Visual displays of data in the form of charts, figures, or graphs, were reported in 61.6% (n = 133) of the articles. The odds ratio was the most commonly reported epidemiological statistic (40.7%, n = 88). P-values and confidence intervals were the most commonly reported results from the use of inferential statistics, appearing in 72.2% (n = 156) and 76.4% (n = 165) articles, respectively. The reporting of more than one level of significance, indicated by a hierarchy of ‘*’ symbols (e.g., *p<*0.10*, *p<*0.05**, *p<*0.01***), was used in 18.1% (n = 39) of the studies. Adjustments for multiple testing were only reported in 5.1% (n = 11) of the studies reviewed. The Pearson’s Chi-Square or Fisher’s Exact statistical test were used in 25.9% (n = 56) of the reviewed articles.

**Table 2:** Frequency of reported types of studies and use of descriptive and inferential statistics (n = 216).

	Count	Percent
Article type (select one)		
Methodological	8	3.7
Substantive	201	93.1
Other^1^	7	3.2
Study Design (select one)		
Observational	177	81.9
Experimental	12	5.6
Systematic Review or Meta-Analysis	17	7.9
Other^2^	10	4.6
Analytic Framework		
Primary Data Collection	38	17.6
Summary Statistics		
Descriptive Statistics	198	91.7
Tables	206	95.4
Charts/Figures/Graphs	133	61.6
Epidemiological Statistics		
Prevalence	37	17.1
Relative Risk	27	12.5
Odds Ratio	88	40.7
Incidence	19	8.8
Mortality	25	11.6
Hazard Ratio	32	14.8
Other^3^		
Statistical Inference		
p-values	156	72.2
Confidence Intervals	165	76.4
*,**,*** p-value reporting used	39	18.1
Multiple Testing	11	5.1
Statistical Tests		
t-test	28	13.0
Pearson’s Chi-square or Fisher’s Exact	56	25.9
Kaplan Meir	5	2.3
Log-rank	2	1.0
Nonparametric (any)	17	7.9
Correlation	34	15.7
Other^4^		

^1^Other article types included program evaluation, evaluation assessment, and a study design overview

^2^Other study designs reported included qualitative studies

^3^Other epidemiological statistics with more than one reported use included sensitivity, specificity, positive predictive value, negative predictive value

^4^Other statistical tests with more than one reported use included the bootstrap, Cronbach’s alpha, Cochran’s Q, Hardy-Weinberg equilibrium test

Frequency of reported use of statistical models in the public health literature are reported in Table 3. We classified all types of logistic regression analyses (including binomial, ordinal, and multinomial) that assumed independent observations into a single category labeled simply as “Logistic Regression.” This was the most commonly reported statistical modeling technique used in the articles reviewed (38.4%, n = 83). Linear regression and Cox Proportional Hazards Regression were reported in 19.4% (n = 42) and 15.3% (n = 33) articles, respectively.

**Table 3:** Frequency of use reported for statistical modeling and related advanced statistical techniques (n = 216).

	Count	Percent
Statistical Modeling, Independent		
Analysis of Variance (ANOVA)	21	9.7
Linear Regression	42	19.4
Logistic Regression	83	38.4
Poisson Regression	16	7.4
Cox Proportional Hazards Regression	33	15.3
Nonlinear Regression	1	<1
Other^1^	22	10.2
Statistical Modeling, Dependent		
General Linear Mixed Model	15	6.9
Generalized Linear Mixed Model	22	10.2
Marginal Models (Generalized Estimating Equations/GEE)	16	7.4
Other^2^	5	2.3
Complex Statistical Modeling Techniques		
Structural Equation Modeling	2	1.0
Mixture Model	1	<1
Latent Class Model	3	1.4
PCA/Factor Analysis	9	4.2
Other^2^	4	1.9
Missing Data		
Casewise Deletion	66	30.6
Mean (Single) Imputation	10	4.6
Multiple Imputation	12	5.6
Other^2^	9	4.2

^1^Other statistical models with more than one reported use included difference in difference, loglinear, ordinal logistic, multinomial logistic, and negative binomial regression

^2^No methods other than those listed were reported more than once

Advanced statistical models that accommodate an independence assumption violation required of classical statistical methods are displayed in Table 3 as dependent statistical models. The general linear mixed model, which assumes a normal distribution, was reported in 6.9% (n = 15) articles, and the generalized linear mixed model, which includes an extension of logistic and Poisson regression models to allow for dependent data, were reported 10.2% (n = 22) of the time. Complex statistical modeling techniques, including structural equation modeling and latent variable models, were reported in less than 5% of the study sample. Missing data was handled most often with casewise deletion (30.6%, n = 66). Multiple imputation was only used in 5.6% (n = 12) of the studies reviewed.

Statistical software packages cited in the reviewed articles is described in Table 4. The most common statistical software package cited as used by study authors was the SAS Software System. STATA was the second most commonly used software package (25.5%, n = 54). R was used in (8.3% n = 18) of the studies.

**Table 4:** Frequency of use reported for statistical software packages (n = 216).

	Count	Percent
Statistical Software		
SAS	75	34.7
SUDAAN	9	4.2
SPSS	18	8.3
STATA	54	25.0
R	18	8.3
Other^1^	32	14.8

^1^Other packages with more than one reported use included ARCGIS, HLM, IVEWARE, MPlus, BUGS

## Discussion

In order to properly and adequately train public health professionals to access scientific publications, it is essential to, at a minimum, be teaching statistical methods actually used and reported in top tier public health journals. Classical statistical frameworks, including hypothesis testing, confidence intervals, and statistical models, are essential and need to be taught in order for a student to read and comprehend what is being published. Our study results show that descriptive statistics were reported in a tabular or graphical format in more than 95% of the articles reviewed. Somewhat surprisingly, when statistical techniques were used, classical statistical modeling techniques were infrequently used, with logistic regression as the most commonly reported type of model applied in the articles reviewed. While these study data only quantify the methods used in the literature, based on its frequent use we advocate for logistic regression to be included in biostatistics education for graduate public health students. It is not specifically mentioned in the current ASPPH competency guidelines for MPH students [4].

Less than half of the studies reviewed mentioned anything about missing data. It is extremely unlikely that missing data is not encountered in the majority of public health research. This lack of reporting about missing data, including attrition, non-response, and dropouts, may reflect a need for journal submission guidelines to require mention of missing data, including its frequency, and how it was addressed in the statistical analysis. About a third of the studies reported using casewise deletion, a relatively outdated and biased approach for analyzing missing data. Missing data is a well-recognized challenge with human subject research. Modern methods for handling missing data (e.g., multiple imputation) were rarely used. This indicates several possible needs. On one hand, in order for newly developing public health professionals to read and understand the limitations of inadequately handling missing data in a statistical analysis, biostatistics education needs to include training on this topic. And on the other, public health professionals may benefit from an introduction to modern methods for handling missing data in a short course or continuing education workshop.

About 18% of studies reported significance testing results with a notation of some variation of the following format: *p < .05, **p < .01, ***p < .001. A result with two asterisks is mistakenly interpreted as more significant than a result with one asterisk [7]. The level of significance in a scientific investigation, also known as alpha (α), is a fixed quantity determined before observing the data. In fact, all that is meaningful is whether or not the p-value is less than alpha. Use of the asterisks notation indicates a possible misunderstanding of p-values and the classical null hypothesis significance testing process used in determining statistical significance [8]. The relatively high frequency of this problematic reporting could be avoided with education and training on appropriate statistical reporting of inferential statistics.

Statistical software is needed to analyze data. SAS and STATA were the two most commonly used packages reported. Exposure to one or both of these packages may be beneficial. Online training courses in statistical methods and statistical software have grown in popularity and may be an option for many working professionals seeking additional training in a format that is manageable with a full time position.

About 82% of studies were observational and less than 6% experimental. As the modern data age continues to evolve, with the increasing use of administrative and other large data sources, it seems plausible to expect more observational data not originally intended for research to become available and used in public health research. Avoiding misuse and ensuring scientific validity of health-related findings from such sources depends on well-educated and trained public health professionals. Although experimental studies remain as the gold standard for enabling causal inference, only a handful were reported. And while there are statistical methods that make causal inference with observational data possible, these approaches were scarcely used in our study sample.

When statistical techniques were used, the vast majority of statistical methods seen in our sample were classical statistical techniques commonly taught in a first or second course in introductory and intermediate statistics. Classical statistics is based on normal theory and rooted in the general linear model (GLM), a framework that includes the three t-tests, linear regression, and ANOVA. The GLM paradigm assumes independence between observations. When this assumption is violated, as is the case with repeated measures data, more advanced statistical techniques are needed to account for the data dependencies that arise. Advanced statistical modeling techniques, including mixed and marginal models, are such methods. However, these techniques, as well as complex statistical modeling techniques such as structural equation modeling and factor analysis, were rarely applied and reported.

The scarce reporting of advanced methods could be an indication that these methods are not of importance or relevance in public health studies. However, since training in these methods has only become available in more recent years, we postulate this may be due to the historic lack of education and training availability on these topics. Many of the advanced statistical techniques rarely observed in our study are methods that were not available in mainstream statistical software ten to twenty years ago. For example, seasoned researchers may not have been exposed to modernized statistical modeling techniques which now available and appropriate for analyzing dependent or multilevel data [9].

Education in modernized statistical methods, including advanced modeling and computationally intensive statistical techniques, is necessary for staying current and implementing new advanced and methods. In addition to solid training in classical statistics, we suggest that graduate public health programs may also benefit from providing advanced biostatistics education and training opportunities to their students. Statistical software and computing power now enables researchers to readily access and make use of advanced statistical methods. Public health professionals may benefit greatly from continuing education training opportunities that provide a structured foray into such methods, coupled with statistical software training to show how to apply these methods to real world data.

### Limitations

Reporting of a statistical method does not necessarily mean its use was appropriate or correct. We did not evaluate the appropriateness or correctness of application. The work presented here is limited to an assessment of statistical methods currently used in the general public health literature. Methods applied in research studies may not be adequate, correct, or appropriate. Previous work estimates that up to 80% of published research is wasted due to poor methods [10]. Our work did not assess these aspects, and instead focused on quantifying which methods were used.

It is also important to note that the language used by authors to describe some statistical methods varied. For example, classical linear regression was referred to in many ways, including fixed-effects regression, linear regression, least-squares regression, and general linear model. In a few cases, the description of statistical methods used was unclear and necessitated group discussion to come to a consensus. Finally, our study is limited to studies accepted for publication. It would be interesting to assess any possible publication bias resulting from statistical methods used in accepted as compared to rejected manuscripts. Since articles were selected only from 2013, the cross-sectional nature of this study limits an ability to consider how the use of statistical methods has changed over time.

## Conclusions

Statistics knowledge is essential for reading and understanding public health research. Review of a random sample of publications from top tier general public health journals showed descriptive statistics and tabular results were reported in more than 95% of the articles. About three quarters of the articles reviewed reported inferential statistics (e.g., p-value, confidence interval). In addition, classic and advanced statistical models were reported in more than a third of the publications. A working knowledge of descriptive and inferential statistics is essential to comprehend, evaluate, and interpret the results for most research studies. Graduate training for public health students and continuing education in biostatistics education for public health professionals are essential for acquiring and maintaining statistics knowledge, as well as continuing to develop new skills as more complex methods are increasingly used and reported.

There is a noticeable lack of an evidence basis to make curricula decisions about biostatistics education. Biostatistics competencies in graduate public health education include developing and cultivating a student’s ability to read and understand the public health scientific literature. However, little is known about the methods used in the literature. The work presented here may be useful to curriculum committees deciding on course and content offerings.

## Supporting information

S1 File This is the study data in an excel file format. (XLSX)
